# Time‐of‐Flight Secondary Ion Mass Spectrometry Revealing the Organocatalyst Distribution in Functionalized Silica Monoliths

**DOI:** 10.1002/open.202400199

**Published:** 2024-09-27

**Authors:** Raoul D. Brand, Julia S. Schulze, Anja Henss, Bernd M. Smarsly

**Affiliations:** ^1^ Institute of Physical Chemistry Justus-Liebig-University Giessen Heinrich-Buff-Ring 17 D-35392 Giessen Germany; ^2^ Center for Materials Research Heinrich-Buff-Ring 16 D-35392 Giessen Germany

**Keywords:** mesoporous materials, supported catalysts, analytical methods •

## Abstract

Hierarchically porous monolithic silica shows promise as a carrier material for immobilized organocatalysts. Conventional analysis usually includes physisorption, infrared spectroscopy and elemental analysis, among others, to elucidate the pore space and degree of functionalization of the material. However, these methods do not yield information about the spatial distribution of the organic species inside the monolithic reactor. In this work, time‐of‐flight secondary ion mass spectrometry has been applied to characterize the surface of organically functionalized silica monoliths. Cross sections of a silica monolith functionalized with 4‐dimethylaminopyridine were analyzed and the results were compared with physisorption and elemental analysis experiments of the same material. This way, insight into the radial distribution of the catalyst could be achieved, which might assist in interpreting the performance of such reactors in heterogeneous flow catalysis.

## Graphical abstract

In this work, porous silica monoliths that have been functionalized for organocatalysis were investigated using time‐of‐flight secondary ion mass spectrometry to reveal the radial catalyst distribution inside the reactor which is usually not obtainable by conventional methods.

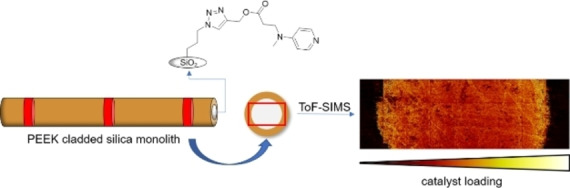

## Introduction

**Figure 1:**
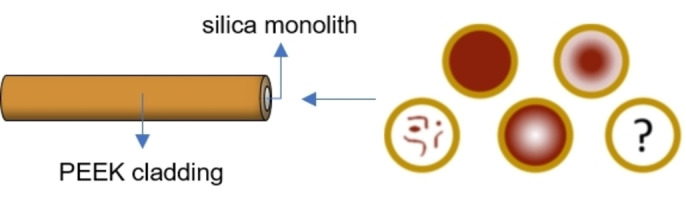
Schematic illustration of possible catalyst distribution patterns inside a cross section of a functionalized silica monolith cladded with polyether ether ketone (PEEK).

Hierarchically meso‐/macroporous silica monoliths represent a promising carrier material for immobilizing catalytically active species such as nanoparticles or organocatalysts.^[1]^ While their macroporous network ensures unhindered mass flow, the mesopores provide the surface area needed to achieve high catalyst loadings. Because of these features, they have been successfully implemented in HPLC, but recently also as reactors in heterogeneous flow catalysis of different organic reactions.[^2^, ^3^] For characterization, commonly different analytical methods are applied. Gas adsorption experiments offer information about the pore space and how it is affected upon functionalization with organic moieties. For example, the mesopore volume and pore size distribution can be obtained by nitrogen physisorption. Additionally, X‐ray or electron tomography represent powerful tools to visualize the pore space through 3D reconstruction with high spatial resolution in the nanometer range, also providing information such as pore connectivity and wall strength.^[4]^ Coupled with XRD or EXAFS, it is also possible to image certain heterogeneous catalysts such as metal nanoparticles on mixed oxides.^[5]^ However, organocatalysts remain elusive to such techniques. The influence of the catalyst distribution inside a reactor plays a critical role in performance optimization as concentration gradients can both directly influence the reaction rate through spatial effects at the catalyst sites but also through heat gradients that influence the reaction rate and can even raise safety concerns if present in large scale reactors. As such, a number of studies, especially on industrially applied catalytic systems, elucidate the topic, however, works on the organocatalyst distribution inside monolithic silica remain underrepresented.[^3^, ^6^] To gain chemical information about the surface in these materials, solid‐state nuclear magnetic resonance (NMR) spectroscopy, thermogravimetric analysis coupled with mass spectrometry (TGA‐MS) as well as diffuse reflectance infrared Fourier transform spectroscopy (DRIFT) and elemental analysis (EA) are routinely applied. Both physisorption and elemental analysis require a significant amount of sample, which, for this kind of material, can be difficult given the size of the monolithic reactors. To reach the minimum sample amount for analysis (up to 50 mg), larger parts of 1–2 cm of the monolith have to be taken because of the high porosity of the material, leading to averaged results without local information which do not appropriately depict the degree of functionalization among the whole length of the monolith. Additionally, these methods only yield information about the longitudinal catalyst gradient and fail to give insight into the radial distribution of the catalyst inside a reactor, as only the bulk material is analyzed and no information about the cross‐section surface is obtained. The latter is of importance to evaluate the performance of the reactor, as it is unclear whether the mass flow through the monolith is indeed ensuring a homogeneous distribution of the catalyst in the functionalization step. As shown in Figure 1, different possible patterns of catalyst distribution inside the cross section of a monolith can be envisioned. Gradients towards the walls or center, as well as irregular patterns caused by channels within the reactor are possible, each limiting the catalytically active surface of the reactor. If large amounts of the material remain inaccessible to functionalization, the resulting flow reactor will be inefficient regarding the portion of the catalytically active material in relation to the sum of the reactor. Likewise, areas with exceedingly high loadings might locally block the pore access for the reaction to take place. Apart from that, without knowledge of the catalyst distribution inside the reactor, the comparability between samples cannot be guaranteed, as experimental results may not necessarily reflect the catalytic activity of the material but rather a disadvantageous distribution pattern. As such, since the distribution of the organocatalyst inside the monolithic reactor cannot be elucidated by conventional methods alone, additional techniques have to be applied to obtain a more detailed picture of the material. In literature, only few examples exist of such experiments, mainly applying energy‐dispersive X‐ray spectroscopy (EDX), with which only a mapping of specific elements is possible, or confocal laser scanning microscopy, which relies on fluorescent probe molecules and is not applicable to reactors cladded by steel or plastics.^[7]^

Time‐of‐flight secondary ion mass spectrometry (ToF‐SIMS) is a surface analysis method that has been used as a versatile tool in material sciences because of its high sensitivity and spatial resolution and ability to identify a large variety of different analytes label‐free.^[8]^ The samples are bombarded with a focused primary ion beam in an ultra‐high vacuum, and the resulting secondary ions are then extracted and analyzed based on their mass‐to‐charge ratio. ToF‐SIMS has been applied, for example, for analyzing solid electrolyte interfaces and biomolecules in osseous tissue, among others.^[9]^ However, there are few examples of investigating organic surface modifications, especially on porous materials such as monolithic silica.^[3][10]^ With this work, in which we elucidate the distribution of the organocatalyst 4‐(Dimethylamino)pyridine (DMAP) inside a silica monolith surrounded by a typical polymer cladding (polyether ether ketone, PEEK), we aim to provide a proof‐of‐concept example that demonstrates the potential of ToF‐SIMS in this research area.

## Results and Discussion

### Characterization of the Carrier Material

**Figure 2:**
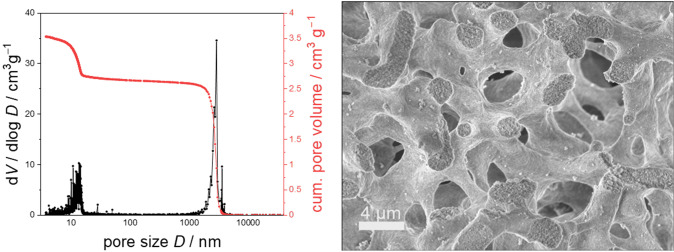
Pore size distribution by mercury intrusion porosimetry (left) and SEM image (right) of the monolithic silica.

The silica‐monoliths used in this study have been synthesized by a modified Nakanishi‐process to obtain a hierarchical meso‐macroporous material. The bimodal pore structure can be seen in the scanning electron microscope (SEM) image shown in Figure 2. The macroporous silica skeleton formed during the sol‐gel process consists of disordered silica struts which themselves possess smaller mesopores introduced during the hydrothermal treatment. Mercury intrusion porosimetry reveals the pore size distribution with the two typical domains ranging from 10–20 nm and 2–4 μm.

For further use as a flow reactor, the silica monoliths were cladded in PEEK by heating both inside a Polytetrafluoroethylene (PTFE) shrinking tube.^[11]^ A tight connection between the monolith and the cladding is essential to prevent channels that could cause the solution to bypass the monolith during application. However, the cladding process also makes further analysis of the functionalized material difficult, as the silica monolith has to be separated from the cladding for conventional analysis (e. g. physisorption), grinding the material to a powder during the procedure.

### Functionalization of the Silica Monolith with DMAP

**Scheme 1:**
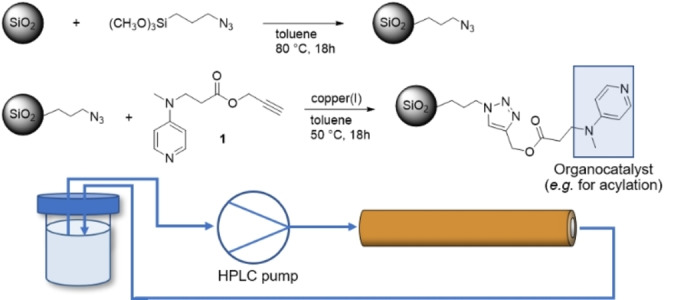
Functionalization of the silica material with the organocatalyst DMAP via copper catalyzed cycloaddition.

**Figure 3:**
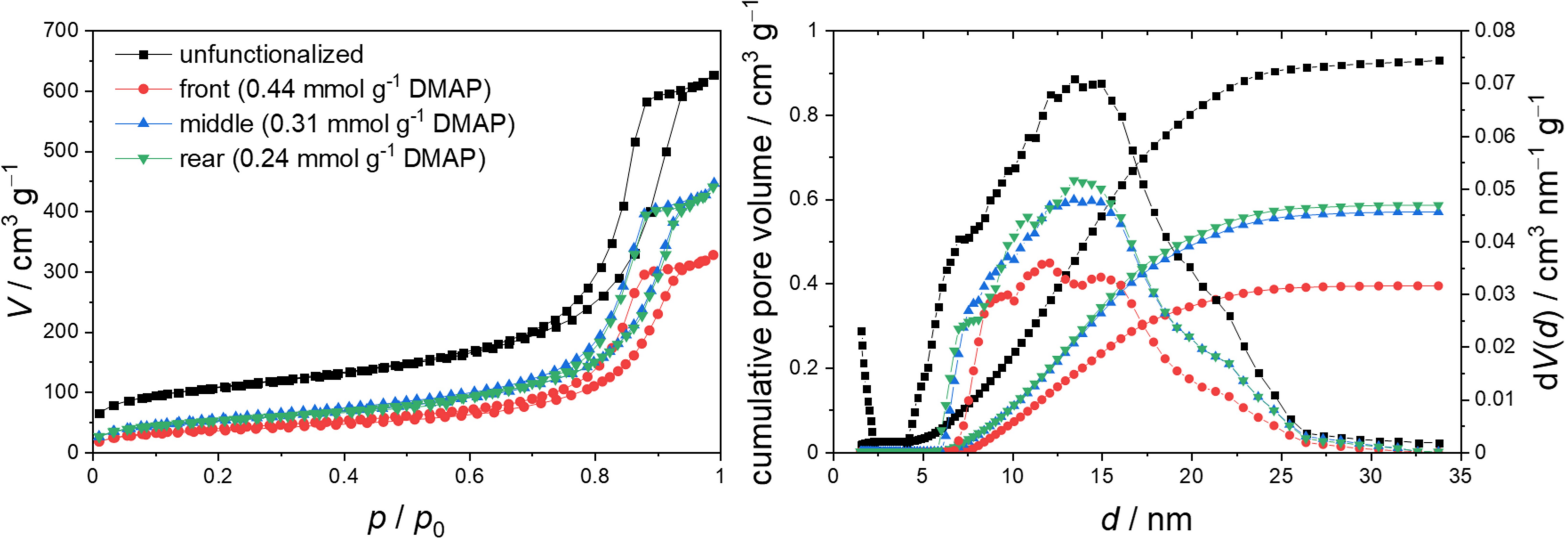
Nitrogen physisorption isotherms (left) and pore size distribution with cumulative pore volume (right) of an unfunctionalized silica monolith (black) and a DMAP functionalized monolith with samples taken from the front (red), middle (blue) and rear part (green).

Functionalization of the silica monoliths was carried out using a DMAP derivative with an alkyne moiety in a copper‐catalysed cycloaddition reaction. For this, the material was first modified with a linker containing the necessary azide group by post‐synthetic grafting with the corresponding alkoxysilane, as seen in Scheme 1. Because the organic moieties would decompose during the cladding process which takes place at elevated temperatures, the functionalization of the silica monolith has to be performed afterwards, within the assembled flow reactor. Reactions were carried out using an HPLC pump in a circular flow setup, with the solution passing through the monolith repeatedly overnight. The functionalized silica monolith‐PEEK reactor was cut crosswise into pieces of circa 3 mm thickness, of which three were later used for the ToF‐SIMS measurements. The other pieces were utilized for nitrogen physisorption analysis, yielding three samples representing the front, middle and rear part of the monolith. The isotherms obtained are shown in Figure 3, compared to the unfunctionalized material. DRIFT spectra of the functionalized monolith indicate an almost complete transformation of the azide group (Figure S1, supporting information.

The shape of the isotherms is of type IV and also features a hysteresis loop as typical of mesoporous silica material of this kind.^[12]^ Upon functionalization, a pronounced decrease in the adsorbed volume can be observed, as the organocatalyst occupies space inside the mesopores. Comparing the three different samples obtained from the monolith, a longitudinal inhomogeneity in the degree of functionalization becomes apparent. The isotherm corresponding to the front part of the monolith (red) is shifted towards lower adsorbed volumes, indicating a larger surface coverage of the organocatalyst in this part of the reactor compared to the middle and rear part (blue and green respectively). To quantify the catalyst loading on the silica monolith elemental analysis has been employed, using the nitrogen content of the sample for the calculation.

The results of the elemental analysis (Figure 3, legend) confirm the functionalization gradient along the monolith, with the front part displaying an increased catalyst loading. While the physisorption isotherms of the middle and rear part show very little difference, elemental analysis suggests an additional decrease in catalyst loading in the rear part. However, the results of the elemental analysis are affected by a higher degree of uncertainty because of the very low number of organic groups compared to the bulk of the material.

### ToF‐SIMS Measurements

**Figure 4:**
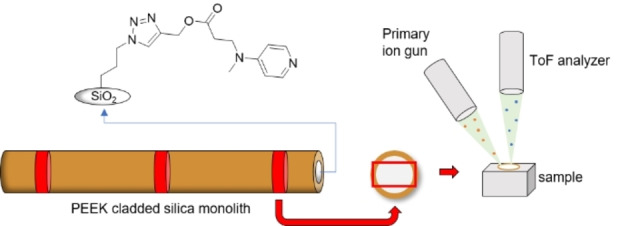
Schematic illustration of the measurement set up and a silica monolith with the ToF‐SIMS measurement window indicated in red (3.2 mm×1.2 mm).

For the ToF‐SIMS measurements, we selected three cross sections from the silica monolith, representing the front, middle, and rear portions as illustrated in Figure 4. The process of sample preparation posed several challenges that required careful consideration. We exercised particular care in choosing cross sections with impeccably smooth surfaces, free from significant cracks, as additional polishing could potentially distort the results by altering the catalyst distribution. Moreover, it was imperative to prevent any contaminations of the PEEK cladding on the silica matrix. However, the most critical challenge stemmed from the insulating nature of the sample, resulting in the accumulation of positive charge on the surface due to the primary ion beam bombardment during the measurement. This accumulation of charge was an obstacle to mapping larger areas (e. g. stage scan of a longitudinal section of the entire monolith), since despite charge compensation by the low‐energy floodgun, signal loss could occur in extreme cases, and in addition the mass resolution was impaired.^[13]^ The interface between the PEEK cladding and the silica proved to be particularly challenging for analysis.

To overcome these issues, we employed a strategic approach. We followed the described method to prepare cross sections. Before mounting on the sample holder, we carefully positioned the samples on a clean glass slide (Figure S2, supporting information). This additional insulation step in combination with the usage of the low‐energy flood‐gun, facilitated a consistent and homogeneous ion extraction field, improving ion yield. Consequently, we achieved a more reliable measurement window of 3.2 mm×1.2 mm, encompassing the majority of the silica material within the cross section and effectively addressing the transition zone between the PEEK cladding on the left and right borders.

**Figure 5:**
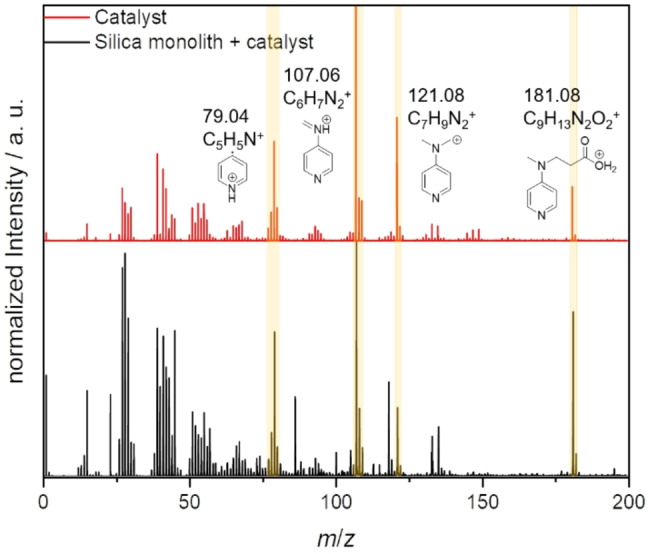
Mass spectra of the catalyst (red) and the functionalized silica monolith (black). Both spectra feature mass signals that can be attributed to the DMAP moiety of the catalyst motif.

**Figure 6:**
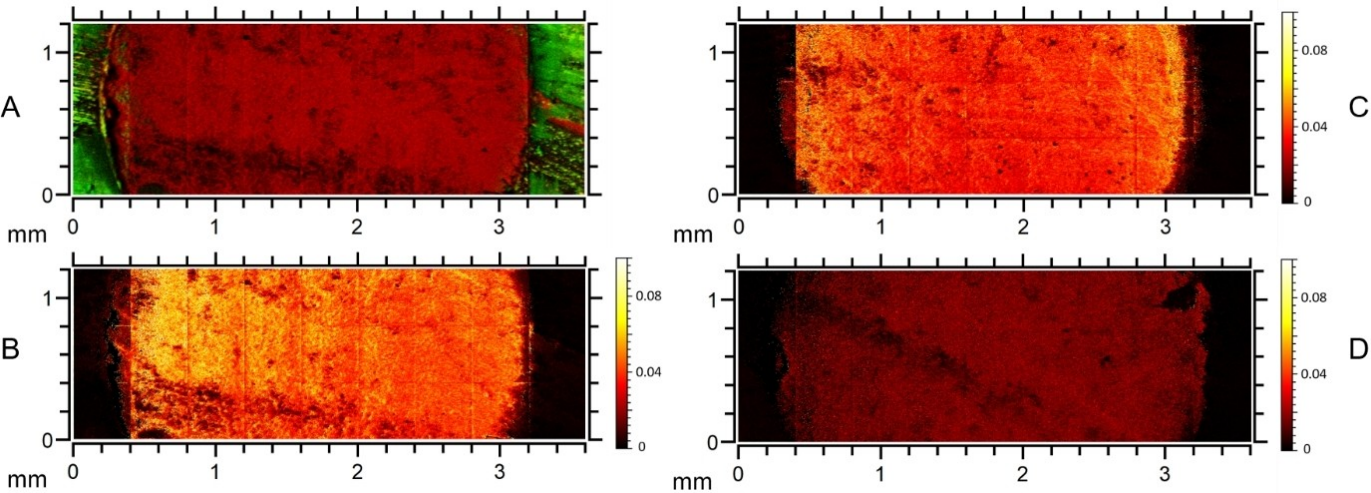
ToF‐SIMS images of different cross sections of a functionalized silica monolith: An overlay of Si^+^ (red) and Na^+^ (green) to visualize silica and PEEK domains, respectively (A); The distribution of a characteristic catalyst fragment in the front (B), middle (C) and rear (D) part of the monolith by the mass signal *m/z* 107, normalized to the total ion count.

Figure 5 shows the mass spectra gained from a reference measurement of the catalyst alone and the functionalized silica monolith. As can be seen, a number of catalyst fragments which stem from the DMAP moiety of the immobilized species can be identified and appear in both samples. For the mapping (Figure 6), the mass signal of *m/z* 107.06, which showed the highest intensity, was chosen and normalized to the total ion count. The PEEK cladding could be identified by the Na^+^ signal, which presumably is caused by residues of the sodium salts used in the production of the polymer.^[14]^

Part A of Figure 6 shows the cross section of the front part of the monolith, which visualizes the two domains of the silica carrier material (red) surrounded by the PEEK cladding (green). Parts B through D depict the distribution of the catalyst in the three different parts of the monolith with the signal of the catalyst fragment normalized to the total ion count, to allow for comparison between the three measurements. The obtained images show that, for the most part, the distribution of the catalyst appears to be homogeneous over the whole silica surface, with no specific pattern of channels or diminishing concentration towards the edges or centre. Additionally, when comparing the catalyst density between the three different samples, a significant decrease in intensity can be observed from the front to the rear part of the monolith (B through D). As mentioned before, this trend of a more strongly functionalized front part of the monolith is also confirmed by physisorption, as the corresponding isotherm shows a significant shift towards lower adsorbed gas volumes. The ToF‐SIMS images also agree with the results of the elemental analysis, showing different catalyst loadings for each part of the monolith.

Besides of some additional catalyst intensity in the area of the PEEK cladding (visible for example in the border regions of B), which is presumably a contamination introduced during the sawing of the monolith, there are also areas of low intensity irregularly distributed throughout the sample, most notable in the cross section of the front part shown in A and B. The black area in the lower half of the cross section can be identified as a larger crack in the silica surface, introduced during the sawing of the monolith. Such strong surface roughness reduces the number of secondary ions reaching the detector when measuring compared to adjacent points. Thus, both the Si^+^ signal (A) as well as the catalyst signal (B) are diminished there. Concludingly, such apparently low catalyst signal is not indicative of a generally inhomogeneous catalyst distribution. If the surface shows less roughness, a more even distribution can be seen as in cross section C (Figure 6). As the used silica monolith is a macroporous material, and the lateral resolution is in the micrometer range, certain inhomogeneities can also be explained by the pore structure of the material, creating intensity differences between adjacent pixels. Indeed, as one pixel equals a distance of 1 μm in the images, the smaller black spots might actually depict some of the macropores, which typically measure 2–4 micrometers (Figure 2), assuming that the spot size of the beam is small enough (less than two microns). A comparison with the SEM images (Figure S3 in the supporting information), which show the macropores present in the surface of the material, also supports this interpretation

. However, further measurements producing high resolution images are required to clarify this issue.

Another factor that needs to be considered when making statements about the catalyst distribution, is thatthe ionization yield of the immobilized catalystmight vary throughout the sample, which is known as the matrix effect. However, since the catalyst concentration changes only slightly between adjacent regions on one sample, the matrix in this case can be considered constant, which allows for a semi‐quantitative or qualitative comparison between the samples, especially because identical measurement conditions and a dose density limit have been maintained. Nevertheless, the matrix effect remains a limitation in this study, and further studies will need to be performed to ensure a truly quantitative comparison.

## Conclusions

Porous carrier materials such as monolithic silica are a topic of interest in the field of heterogeneous catalysis, with characterization methods with respect to the catalyst loading typically comprising physisorption, infrared spectroscopy and elemental analysis among others. Such methods, however, do not yield spatially resolved surface information, which is necessary to investigate the radial distribution of catalyst inside functionalized monolithic materials to ascertain a homogeneous distribution and maximize efficiency for the application as a flow reactor. Our study shows that ToF‐SIMS can be applied to these kind of samples to complement traditional methods and help elucidate the degree of functionalization both longitudinally and radially to evaluate the homogeneity on the scale of micrometers. For our project, a silica monolith functionalized with the organocatalyst DMAP was divided into multiple cross sections which were analyzed individually, creating images of the catalyst distribution inside the reactor. The ToF‐SIMS measurements revealed a largely homogeneous radial distribution, whereas the catalyst loading decreases longitudinally along the monolith, further confirmed by physisorption and elemental analysis. A major difficulty in sample preparation, besides charging effects, is ensuring an even and smooth surface, with the silica material being prone to cracks creating significant surface roughness. This can be detrimental to the secondary ion yield, thus creating areas with decreased intensity which do not necessarily reflect the actual catalyst distribution. Further research is planned to apply the method to other, more complex organocatalysts. Additionally, since this method can be applied to the complete reactor, a post mortem analysis becomes feasible to investigate possible changes of the functionalized surface or deposition of side products upon usage in heterogeneous catalysis.

## Experimental Section

### Materials

The chemicals sodium azide ≥99 %, (3‐chloropropyl)trimethoxysilane (≥97 %), bromotris(triphenylphosphine)copper(I) (98 %), 4‐(methylamino)pyridine (98 %), tetrabutylammonium iodide (98 %) and urea (99.5 %) were purchased from Merck/Sigma. N,N‐diisopropylethylamine (99.5 %) and ethylenediaminetetraacetic acid disodium salt (99 %) were purchased from Carl Roth. Propargyl acrylate (96 %, stabilized with 200 ppm BHT) was purchased from Alfa Aesar. Tetramethoxysilane (99 %) was purchased from Acros and polyethylene glycol (average Mn 10.000) from Fluka. Solvents were purchased from VWR as HPLC grade or distilled before use.

#### Preparation of the DMAP Functionalized Silica Monolith

The synthesis of the DMAP functionalized silica monolith was performed as described in a previous publication.^[15]^

#### 3‐(azidopropyl)trimethoxysilane

1.76 g (27.1 mmol) sodium azide and 0.201 g (0.624 mmol) tetrabutylammonium iodide were dried overnight under vacuum. Both were suspended in 15 mL acetonitrile under argon and 1 mL of (3‐chloropropyl)trimethoxysilane was added before stirring at 90 °C for five days. Afterwards, the suspension was filtrated and the solvent removed under reduced pressure. The crude product was dissolved in dry pentane and solid residues again removed by filtration. The process was repeated several times until no solid residues precipitated and 0.542 g of a colorless liquid were obtained (48 % yield), with a purity of 93 % as determined by ^1^H‐NMR. ^1^H‐NMR (400 MHz, CDCl3): δ (ppm)=3,51 (s, 9H, RSi(OCH3)3, 3,20 (t, 2H, R−CH2−N3), 1,64 (p, 2H, R−CH2−CH2−N3), 0,63 (t, 2H, Si−CH2−R)

#### DMAP Derivative

0.251 g (2.23 mmol) of N‐methylaminopyridine was mixed with 1 mL (9.05 mmol) of propargyl acrylate and stirred at 90 °C for two hours. Excess propargyl acrylate was then removed by distillation under reduced pressure (90 °C, 10 mbar) and the crude product was purified by column chromatography (silica gel, DCM/MeOH 10 : 1) to receive 0.358 g (1.64 mmol) of a yellow liquid (71 % yield). ^1^H‐NMR (400 MHz, CDCl3): δ (ppm)=8,16 (d, 2H, pyridine), 6,45 (d, 2H, pyridine), 4,61 (d, 2H, R−CH2−COOR), 3,65 (d, 2H, R−CH2−CH2−COOR), 2,93 (s, 3H, R−N−CH3), 2,58 (t, 2H, RCH2−C−CH3), 2,42 (t, 1H, RCH2−C−CH3)

#### Synthesis of the Silica Monoliths

1.200 g polyethylene glycol 10.000 and 0.900 g urea were dissolved in 10 mL acetic acid (0.01 M) and stirred at room temperature for 35 minutes. After cooling in an ice bath, 5.6 mL tetramethoxysilane were added and the solution was stirred for another 20 minutes at 0 °C. After warming to room temperature again, the solution was transferred to stainless steel tubes and aged at 22.5 °C in a water bath for 22 hours. The obtained silica monoliths were then placed in a urea solution (9 g urea in 100 mL 0.01 M acetic acid) and placed in a furnace for hydrothermal treatment (heating to 95 °C over 12 hours and holding the temperature for another 15 hours). The silica monoliths were then placed in methanol and shaken on a laboratory shaker for five days during which the methanol was replaced three times. Subsequently, the monoliths were placed in a furnace for calcination (heating to 330 °C over 10 hours and holding the temperature for another 15 hours.). For the cladding process, the monoliths were put into a PEEK tube surrounded by a PTFE shrink tube and heated to 362 °C.

#### Functionalization of the Silica Monolith

The silica monolith was flushed with toluene for 15 minutes before a solution of 42.4 μL (0.055 mmol) 3‐(azidopropyl)trimethoxysilane in toluene was pumped through the monolith repeatedly over 19 hours at 80 °C with a flowrate of 0.01 mL s^−1^. Afterwards, the monolith was flushed again with toluene for another 20 minutes. For the click reaction, a solution containing 0.160 mmol of the DMAP derivative, 0.267 mmol N,N‐diisopropylethylamine and 12.3 μmol bromotris(triphenylphosphine)copper(I) in toluene was pumped through the monolith repeatedly over 19 hours at 50 °C with a flowrate of 0.01 mL s^−1^. To remove residual copper salt, the monolith was flushed with a 5 *w*% solution of ethylenediaminetetraacetic acid disodium salt in water.

### ToF‐SIMS Measurements

The measurements were carried out with a TOF.SIMS 5‐100 and a M6 Hybrid instrument (IONTOF GmbH), equipped with a 30 kV Bi cluster primary‐ion gun for analysis, a dual‐source column (DSC) and a gas cluster source (GCIB) for depth profiling. Spectra were recorded in the spectrometry mode of the LMIG (bunched, FWHM m/Δm=2600 @m/z=27.02 (C_2_H_3_^+^) at the silica monolith and FWHM m/Δm=7660 @m/z=27.02 (C_2_H_3_^+^) for the pure catalyst) and all purpose mode of the analyser. Stage scans were done by stitching patches of 400×400 μm^2^ and 3 scans in total. The raster size was 3600×1200 pixels. Dose density for all stage scans was 6.2×10^11^ ions/cm^2^. For all measurements, the cycle time was 100 μs and positive polarity was used. The spectra were calibrated to H^+^, C^+^, CH_3_^+^, C_2_H_5_^+^, Cs^+^ Data were evaluated with SurfaceLab 7.3 (IONTOF GmbH). If not stated differently, the samples were electrically isolated from the sample holder and measured with electron neutralization of the flood gun.

### Other Characterization Methods

Nitrogen physisorption experiments were performed using a “Quadrasorb evo” by Quantachrome Instruments at a temperature of 77 K. Pore size distributions were calculated using an NLDFT kernel (N_2_ at 77 K on silica, cyldinrical pores, adsorption branch) provided by the Quantachrome software ASiQwin.

Mercury intrusion porosimetry was performed using a Pascal 140 and 440 porosimeter by Thermo Fisher Scientific in a pressure range of 0–400 mPa.

For scanning electron microscopy, the sample was sputter coated with platinum and measured with a Zeiss Merlin (acceleration voltage of 2.00 kV, current of 113 pA).

A CHN‐analyzer Flash EA‐1112 (Thermo Scientific) was used for elemental analysis.

## Conflict of Interests

The authors declare no conflict of interest.

1

## Supporting information

As a service to our authors and readers, this journal provides supporting information supplied by the authors. Such materials are peer reviewed and may be re‐organized for online delivery, but are not copy‐edited or typeset. Technical support issues arising from supporting information (other than missing files) should be addressed to the authors.

Supporting Information

## Data Availability

The data that support the findings of this study are available from the corresponding author upon reasonable request.
