# Supplementary material for: Time‐of‐Flight Secondary Ion Mass Spectrometry Revealing the Organocatalyst Distribution in Functionalized Silica Monoliths
Source: ChemistryOpen. 2024 Sep 27;13(11):e202400199. doi: 10.1002/open.202400199 (PMC12056919; doi:10.1002/open.202400199)
Supplement: Supplementary file 1 — Supporting Information [file OPEN-13-e202400199-s001.pdf]

ChemistryOpen

Supporting Information

Time-of-Flight Secondary Ion Mass Spectrometry Revealing the Organocatalyst Distribution in Functionalized Silica Monoliths

Raoul D. Brand, Julia S. Schulze, Anja Henss, and Bernd M. Smarsly*

Supporting Information

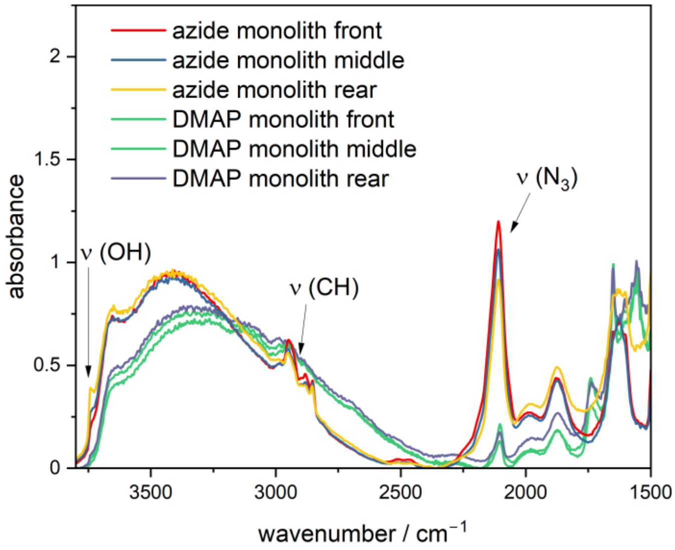

Figure S1: Diffuse reflectance Fourier transform infrared spectra of an azide precursor monolith and the fully functionalized DMAP monolith. The intensity of the characteristic vibrational band of the azide group (2110 cm^{-1}) is diminished greatly upon reaction, indicating an almost complete transformation.

The prepared cross sections of the silica monolith were carefully positioned on a glass slide by using conductive adhesive copper tape and then mounted on the sample holder.

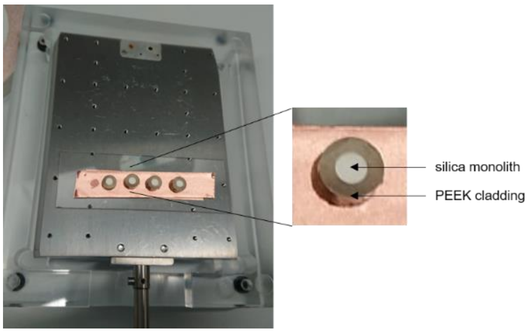

Figure S2: Picture of the prepared cross sections mounted on the sample holder.

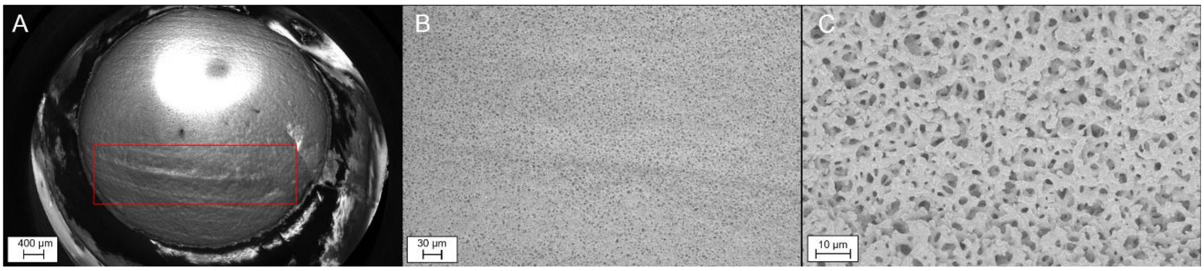

Figure S3: SEM images of the cross section of the front part of the monolith (image A and B in Figure 7 of the main manuscript). Picture A shows the silica monolith as well as the surrounding PEEK cladding, with a larger edge presumably caused by the sawing process marked in the red square. Through higher magnification (B and C) the macroporous structure of the material becomes apparent.
